# Paroxysmal Dyskinesias in Paediatric Age: A Systematic Review

**DOI:** 10.3390/jcm14175925

**Published:** 2025-08-22

**Authors:** Giulia Pisanò, Martina Gnazzo, Giulia Sigona, Carlo Alberto Cesaroni, Agnese Pantani, Anna Cavalli, Susanna Rizzi, Daniele Frattini, Carlo Fusco

**Affiliations:** 1 Child Neurology and Psychiatry Unit, Dipartimento Materno-Infantile, Presidio Ospedaliero Santa Maria Nuova, AUSL-IRCCS di Reggio Emilia, 42123 Reggio Emilia, Italy; giuliapisan@gmail.com (G.P.); martignazzo@hotmail.it (M.G.); giuli.sigona@gmail.com (G.S.); agnese.pantani@ausl.re.it (A.P.); anna.cavalli@ausl.re.it (A.C.); susanna.rizzi@ausl.re.it (S.R.); daniele.frattini@ausl.re.it (D.F.); carlo.fusco@ausl.re.it (C.F.); 2 Laboratorio di Neurofisiologia Pediatrica, Dipartimento Materno-Infantile, Azienda USL-IRCCS di Reggio Emilia, 42123 Reggio Emilia, Italy

**Keywords:** paroxysmal dyskinesia, paediatric movement disorders, episodic dystonia

## Abstract

**Background:** Paroxysmal dyskinesias (PDs) are rare, episodic movement disorders characterized by sudden and involuntary hyperkinetic motor events. In paediatric populations, their diagnosis is often complicated by clinical overlap with epilepsy and other neurological conditions. Genetic underpinnings have increasingly been recognized as key to understanding phenotypic heterogeneity and guiding treatment. **Objectives**: This systematic review aims to provide a comprehensive overview of paediatric PD, with a focus on genetic aetiologies, clinical features, subtype classification, and therapeutic approaches, including genotype–treatment correlations. **Methods**: We systematically reviewed the literature from 2014 to 2025 using PubMed. Inclusion criteria targeted paediatric patients (aged 0–18 years) with documented paroxysmal hyperkinetic movements and genetically confirmed or clinically suggestive PD. Data were extracted regarding demographics, dyskinesia subtypes, age at onset, genetic findings, and treatment efficacy. Gene categories were classified as PD-specific or pleiotropic based on functional and clinical features. **Results**: We included 112 studies encompassing 605 paediatric patients. The most common subtype was Paroxistic Kinesigenic Dyskinesia (PKD). Male sex was more frequently reported. The mean onset age was 5.99 years. A genetic diagnosis was confirmed in 505 patients (83.5%), involving 38 different genes. Among these, *PRRT2* was the most frequently implicated gene, followed by *SLC2A1* and *ADCY5*. Chromosomal abnormalities affecting the 16p11.2 region were identified in ten patients, including deletions and duplications. Among the 504 patients with confirmed monogenic variants, 390 (77.4%) had mutations in PD-specific genes, while 122 (24.2%) carried pleiotropic variants. Antiseizure drugs—particularly sodium channel blockers such as carbamazepine and oxcarbazepine—were the most frequently reported treatment, with complete efficacy documented in 59.7% of the studies describing their use. **Conclusions**: Paediatric PDs exhibit significant clinical and genetic heterogeneity. While *PRRT2* remains the most common genetic aetiology, emerging pleiotropic genes highlight the need for comprehensive diagnostic strategies. Sodium channel blockers are effective in a subset of genetically defined PD, particularly *PRRT2*-positive cases. Patients with pathogenic variants in other genes, such as *ADCY5* and *SLC2A1*, may benefit from specific therapies that can potentially change their clinical course and prognosis. These findings support genotype-driven management approaches and underscore the importance of genetic testing in paediatric movement disorders.

## 1. Introduction

Paroxysmal dyskinesias (PDs) are rare hyperkinetic movement disorders characterized by sudden, brief and recurrent episodes of involuntary movements, such as dystonia, chorea or athetosis, without loss of consciousness. These episodes are self-limited and may occur spontaneously or in response to specific triggers, distinguishing PD from other movement disorders and epileptic syndromes [1,2,3].

PDs are classically categorized into three main types based on their triggering factors: paroxysmal kinesigenic dyskinesia (PKD), precipitated by sudden voluntary movements; paroxysmal non-kinesigenic dyskinesia (PNKD), triggered by factors such as alcohol, caffeine or fatigue; and paroxysmal exercise-induced dyskinesia (PED), associated with prolonged exertion. A fourth, less common type, paroxysmal hypnogenic dyskinesia (PhD), occurs during sleep [4,5,6].

In paediatric populations, PDs typically present with an average age of onset between 3.9 and 8.8 years and show a male predominance. Dystonia is the most frequently reported symptom, followed by choreoathetosis [6]. Familial forms usually begin in childhood, whereas acquired forms tend to have later onset [7]. Despite clinical classification, significant phenotypic and genetic overlap exists among subtypes [2,6].

From a genetic perspective, PDs exhibit considerable heterogeneity. Mutations in *PRRT2*, *PNKD*, *SLC2A1* and *KCNMA1* genes have been implicated across PD subtypes [5,6,8]. *PRRT2* mutations are the most prevalent and account for approximately 35% of all cases. These mutations are associated with a spectrum of paroxysmal disorders including PKD, benign familial infantile seizures and migraine [9,10]. The most common variant, *c.649dupC*, has been identified in several families using exome sequencing and linkage analysis [11]. Functionally, *PRRT2* encodes a protein that interacts with SNAP-25, regulating synaptic transmission and neuronal excitability [9].

Genotype–phenotype correlations are imperfect but clinically informative. For example, *PRRT2*-positive PKD is associated with earlier onset, bilateral symptomatology and higher attack frequency, as well as a more favourable response to carbamazepine compared to *PRRT2*-negative cases [12]. Similarly, *SLC2A1* mutations underlie some PED cases and may respond to ketogenic dietary interventions [2,6].

The diagnosis of PD in children is challenging due to symptom overlap with epilepsy, psychogenic movement disorders, and metabolic conditions. Electroencephalography (EEG) and, more importantly, next-generation genetic testing are essential for accurate differential diagnosis [5]. A combined approach using detailed clinical assessment and genetic screening is currently recommended [2,6].

Therapeutic strategies for paediatric PD are subtype-specific. PKD generally responds well to low doses of antiepileptic drugs (AEDs), such as carbamazepine and oxcarbazepine, with complete symptom resolution reported in some series [13,14]. PNKD and PED management focuses on identifying and avoiding known triggers [5]. Additional therapies such as acetazolamide, clonazepam, ketogenic diet and botulinum toxin have been employed in selected cases [3,6,15]. For refractory cases, surgical options may be considered [3].

Overall, the prognosis of paediatric PDs, particularly those associated with *PRRT2* and *PNKD* mutations, is favourable, with many children experiencing remission in adolescence or adulthood [3]. However, the clinical and genetic complexity of these disorders necessitates ongoing research to optimize diagnostic accuracy and individualize therapy.

This review aims to provide a comprehensive overview of paroxysmal dyskinesias in the paediatric population, with a specific focus on their genetic underpinnings, clinical features and treatment options. We will explore and examine recent advances and emerging trends in the understanding of these conditions.

## 2. Materials and Methods

This systematic literature review was conducted and reported in accordance with the Preferred Reporting Items for Systematic Reviews and Meta-Analyses (PRISMA) guidelines [16]. The study protocol was prospectively registered on PROSPERO (CRD420251050698).

This scoping review, conducted following the Joanna Briggs Institute methodology, examined literature published between 2014 and 2025 on paediatric paroxysmal dyskinesias. A systematic search was performed in PubMed in April 2025 using the following combination of keywords: (‘paroxysmal dyskinesia’ OR ‘paroxysmal movement disorder’ OR ‘episodic dystonia’ OR ‘paroxysmal chorea’ OR ‘transient dystonia’ OR ‘paroxysmal hyperkinetic’ OR ‘intermittent dyskinesia’ OR ‘paroxysmal motor event’) AND ‘paediatric’. No automated filters (e.g., age, language) were applied in the database. Studies were screened manually according to predefined inclusion and exclusion criteria.

The quality of the included studies was assessed using the Newcastle–Ottawa Quality Assessment Scale. Studies were categorized as having low (≥7 stars), moderate (5–6 stars), or high (≤4 stars) risk of bias, with a maximum score of 9 stars. Two independent reviewers (MG and GP) conducted the quality assessments, and any discrepancies were resolved through discussion and consensus.

Titles and abstracts were independently screened by two reviewers (GP and MG). Full-text articles were then assessed for eligibility using predefined inclusion and exclusion criteria. Disagreements were resolved through discussion and consensus. No automation tools were used.

### 2.1. Inclusion Criteria

We included studies meeting the following criteria:

- Population: paediatric patients aged 0 to 18 years.
- Phenotype: presence of hyperkinetic involuntary movements (including chorea, dystonia, ballism, myoclonus, or combinations) with a paroxysmal course, defined by:
  ○ Sudden onset and limited duration of episodes (ranging from seconds to hours);
  ○ Intermittent or recurrent occurrence over time;
  ○ Return to baseline or relatively stable neurological status between episodes.
- Diagnosis: a recognized clinical or genetic diagnosis associated with paroxysmal dyskinesia (e.g., *PRRT2*, *PNKD*, *SLC2A1*, *GNAO1*, *ADCY5*).
- Phenotypic description: clear documentation or inference of a paroxysmal pattern, even if the term “paroxysmal” is not explicitly used.
- Publication language: studies published in English or other languages with an English abstract available.
- Study type: we considered case reports, case series, cohort studies reporting relevant clinical data.
- Multiple patients or family descriptions: in cases describing multiple patients (either distinct or within the same family), only individuals with childhood-onset were included. In cases where families or groups were reported, paediatric individuals were included if they were individually described with adequate clinical detail, ensuring a clear distinction from other family members or cases.

### 2.2. Exclusion Criteria

We excluded studies with the following characteristics:

- Descriptions of continuous or non-paroxysmal hyperkinetic movements (e.g., progressive dystonias, persistent dyskinesias).
- Neurological conditions without a paroxysmal course of movement disorders (e.g., dyskinetic cerebral palsy, static or neurodegenerative encephalopathies).
- Studies focusing exclusively on adult patients (>18 years)
- Articles lacking sufficient clinical detail to assess the presence or absence of a paroxysmal movement pattern.

For the “age of onset” column, the following approximations were used: early infancy = 0.25 years; infancy = 0.5 years; adolescence = 14.5 years.

For the genetic analysis, we included only patients for whom a specific gene mutation was explicitly reported with a patient count. Cases with chromosomal abnormalities were considered separately unless associated with a confirmed monogenic variant.

The following variables were systematically extracted: age at onset, sex, dyskinesia subtype (PKD, PNKD, PED, PHD), gene or chromosomal anomaly involved, treatment type and response. When only qualitative age descriptors were available (e.g., ‘infancy’), we applied standardized numeric conversions. No data were extracted on funding sources from individual studies.

## 3. Results

### 3.1. Flow Chart of Included Studies

**Figure 1:**
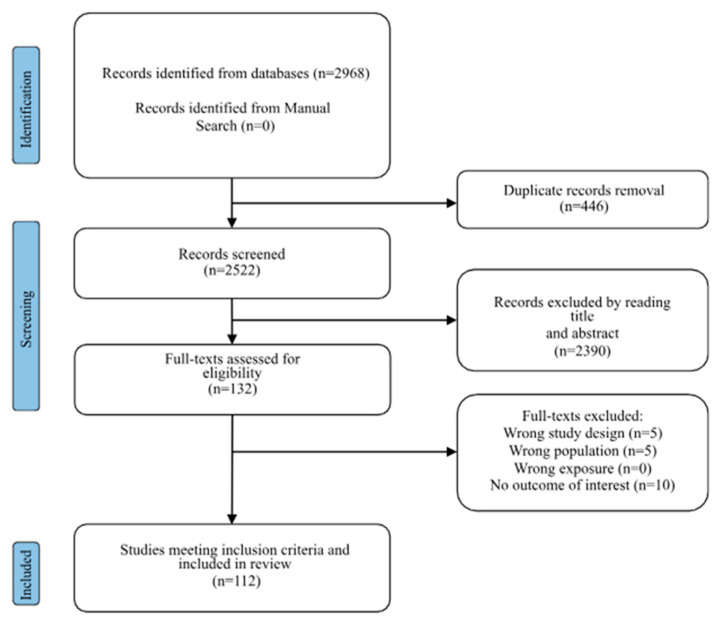
Prisma flow-chart describing the study selection process. Here are references of included articles [2,6,10,17,18,19,20,21,22,23,24,25,26,27,28,29,30,31,32,33,34,35,36,37,38,39,40,41,42,43,44,45,46,47,48,49,50,51,52,53,54,55,56,57,58,59,60,61,62,63,64,65,66,67,68,69,70,71,72,73,74,75,76,77,78,79,80,81,82,83,84,85,86,87,88,89,90,91,92,93,94,95,96,97,98,99,100,101,102,103,104,105,106,107,108,109,110,111,112,113,114,115,116,117,118,119,120,121,122,123,124].

Search results are summarized in the Prisma Flow Chart in Figure 1 (PRISMA 2020 checklist in Supplementary Figure S1). Initially, 2968 studies were identified through the search strategy from the databases. After removing duplicates, 2522 studies remained. Following the title and abstract screening, 132 studies were assessed for eligibility.

Finally, we included 112 studies in the systematic review (see Supplementary Table S1).

### 3.2. Ratings of Study Quality and Risk of Bias

According to the study’s quality ratings, 97 studies were rated as good quality, 9 as moderate quality and 6 as poor quality. Since most of the studies included in this review were of moderate or good quality, the risk of bias from them was low. Three researchers independently extracted data on the variables from the studies.

### 3.3. Population Characteristics

The 112 studies encompassed a total of 605 patients.

#### 3.3.1. Gender Data

Gender information was available for 476 out of 605 patients, as reported in the included studies. Among these, 302 were male (63.4%) and 174 were female (36.6%). Gender data were missing for the remaining 129 patients. Table 1 summarizes the numerical and percentage distribution by gender.

#### 3.3.2. Age at Onset

Data on the mean age at onset were available in 108 studies (601 patients). After standardizing all reported values to years, the mean age at onset across studies was 5.99 years, with a median of 5.00 years. The age at diagnosis does not take into account the diagnostic delay that often occurs for individuals with these movement disorders. The youngest reported mean age was 0.027 years (approximately 10 days), while the oldest was 17 years. Age values originally expressed in days or months were converted to years to allow consistent statistical analysis (see Table 2). In addition, qualitative descriptors were standardized as follows: “early infancy” was coded as 0.25 years, “infancy” as 0.5 years, and “adolescence” as 14.5 years.

### 3.4. Distribution of Dyskinesia Subtypes

Among the paediatric patients reported in the included studies, three major subtypes of paroxysmal dyskinesia were identified: Paroxysmal Kinesigenic Dyskinesia (PKD), Paroxysmal Non-Kinesigenic Dyskinesia (PNKD) and Paroxysmal Exercise-induced Dyskinesia (PED). A total of 343 cases of PKD (56.8%), 142 cases of PNKD (23.5%) and 119 cases of PED (19.7%) were documented. The subtype was not mutually exclusive in all studies, and some reports included patients with multiple overlapping features. Data on subtype classification were available for the majority of patients; entries with missing information were excluded from percentage calculations. Cases originally reported with non-standard nomenclature were classified post hoc according to the most consistent clinical features described by the authors. Studies reporting mixed or ambiguous classifications were carefully reviewed and patients reassigned to the most appropriate subtype whenever possible. These findings are summarized in Table 3.

### 3.5. Paroxysmal Hypnogenic Dyskinesia (PHD) and Nocturnal Trigger in PNKD

In our cohort, Paroxysmal Hypnogenic Dyskinesia (PHD) was explicitly identified as a distinct clinical entity in only two studies, encompassing a total of three patients [52,79]. Notably, the case described by Almeida et al. [52] was associated with brachytelephalangic chondrodysplasia punctata. In the video available in the article [52], we note how it is particularly difficult to distinguish a paroxysmal nocturnal dyskinesia from a nocturnal frontal lobe seizure; an electroencephalographic recording of the episode is essential in order to resolve diagnostic doubts.

These cases lacked consistent genetic characterization.

Conversely, a subset of 25 patients originally classified under Paroxysmal Non-Kinesigenic Dyskinesia (PNKD) exhibited a prominent nocturnal trigger, such as onset during sleep, awakening, or falling asleep. For the purpose of this analysis, these cases were reclassified as PHD based on their clinical features [36,39,45,52,60,63,76,79,98,100,109,117]. Among this reclassified group, genetic variants were identified in 22 patients, including 13 with *ADCY5* mutations (across five studies), 4 with *NACC1*, 2 with *SLC16A2*, and one each with *PRRT2*, *ATP1A3,* and *DNML1*. The remaining three patients had no identifiable genetic cause.

Details are summarised in Table 4.

### 3.6. Genetic Findings

Among the 605 paediatric patients identified, 505 (83.5%) had a genetically confirmed diagnosis, while for 100 patients (16.5%) genetic data were incomplete, inconclusive or unavailable.

Among patients with a genetically confirmed diagnosis, a total of 504 monogenic variants were identified, corresponding to 38 distinct genes. These reflect the cases for which specific molecular findings were available and extractable from the included studies, accounting for 82.0% of the overall cohort. The most frequently reported gene mutation involved PRRT2 (46.29%), followed by SLC2A1 (16.21%) and ADCY5 (5.66%). The full distribution is summarized in Table 5.

Chromosomal abnormalities were reported in 10 patients, all involving the 16p11.2 region and including both deletions and duplications. Notably, one of these patients [97] also carried a PRRT2 mutation. The full distribution is summarized in Table 6.

One patient was reported with brachytelephalangic chondrodysplasia punctata (CDPX1), a rare X-linked skeletal dysplasia, presenting with a paroxysmal non-kinesigenic dyskinesia (PNKD) phenotype.

### 3.7. Gene Categories

To explore the genetic heterogeneity of paediatric paroxysmal dyskinesias (PDs), all genes identified in the included studies were categorized into two main functional groups: PD-specific genes and pleiotropic genes, in accordance with previous classifications [2,125]. PD-specific genes were defined as those predominantly associated with isolated paroxysmal movement disorders, typically without major comorbidities such as epilepsy, intellectual disability or neurodevelopmental delay. These genes are characteristically linked to well-defined PD subtypes (e.g., PKD, PED), with relatively consistent clinical features and often favourable outcomes. In contrast, pleiotropic genes were defined as those associated with broader neurodevelopmental or epileptic syndromes, where paroxysmal dyskinesia may be one of multiple neurological manifestations. These genes are commonly implicated in developmental and epileptic encephalopathies (DEEs), congenital syndromes or complex movement disorders with continuous or combined hyperkinetic signs. Representative examples include *GNAO1*, *SCN8A*, *FOXG1* and *SYNGAP1*.

**Figure 2:**
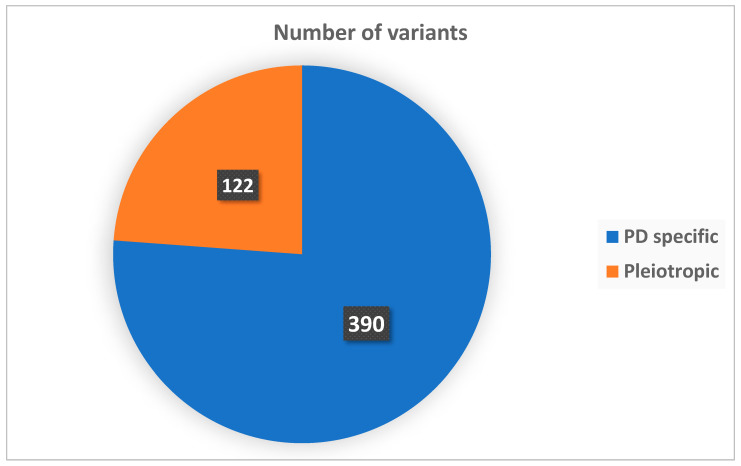
Distribution of genes by functional category.

Based on this classification, 390 variants (76.2%) were mapped to PD-specific genes (e.g., *PRRT2*, *SLC2A1*, *ADCY5*, *KCNA1*, *KCNMA1*, *TMEM151A*), while 122 variants (23.8%) involved pleiotropic genes (e.g., *GNAO1*, *SCN8A*, *FOXG1*, *SYNGAP1*). These proportions reflect the distribution of 512 gene-level diagnoses identified among the 504 patients with confirmed monogenic variants. The complete distribution is illustrated in Figure 2.

### 3.8. Temporal Trends in Genetic Complexity

**Figure 3:**
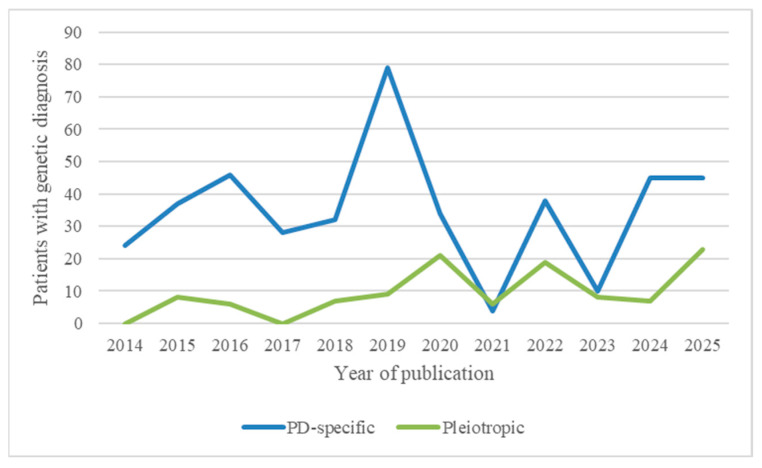
Temporal distribution of genetically confirmed paediatric paroxysmal dyskinesia cases by gene category.

An analysis of the literature over time reveals a shift in the genetic understanding of paediatric paroxysmal dyskinesias. In earlier reports, most patients carried mutations in paroxysmal dyskinesia-specific genes such as *PRRT2*, *PNKD* and *SLC2A1*, typically associated with isolated phenotypes and favourable treatment responses. However, in more recent years, the advent of next-generation sequencing (NGS) technologies has led to the increasing identification of pleiotropic genes such as *GNAO1*, *SCN8A*, *FOXG1*, and *SYNGAP1*, known to cause developmental and epileptic encephalopathies (DEEs) or complex neurodevelopmental syndromes. Figure 3 illustrates the proportional increase in pleiotropic gene-associated cases over the past decade.

### 3.9. Pharmacological Treatment Summary

Due to the heterogeneity in treatment reporting and the frequent lack of patient-level therapeutic data, the analysis was conducted at the study level, considering each publication as a single observational unit.

Among the 112 studies included in the review, 97 (86.6%) reported at least one pharmacological intervention. Of these, 78 studies (80.4%) described the use of antiseizure medications (AEDs).

Beyond AEDs, other pharmacological strategies were documented: the ketogenic diet (KD) in 16 studies (16.5%), dopaminergic therapy (L-dopa/carbidopa) in five (5.2%), baclofen and trihexyphenidyl in four each (4.1%), acetazolamide in three (3.1%), caffeine in three (3.1%), deep brain stimulation (DBS) in three (3.1%) and botulinum toxin in two (2.1%). Other treatments—including thiamine, diazepam, benzodiazepines, flunarizine, tetrabenazine, lisdexamfetamine, gabapentin, clonidine, clonazepam, antioxidants, lomerizine, mitochondrial support, and low-valine dietary therapy—has been reported (1.0%).

Most commonly AEDs used were sodium channel blockers such as carbamazepine (CBZ) or oxcarbazepine (OXC), mainly used in patients with a mutation in the PRRT2 gene. CBZ/OXC treatment was specifically reported in 67 studies (69.1%). Among these, 40 studies (41.2%) described complete symptom resolution, while three (3.1%) reported partial benefit and three (3.1%) no clinical effect. In 21 studies (21.6%), the response to CBZ/OXC was not clearly documented. Genes associated with suboptimal or variable response included *DNML1*, *ADCY5*, *ANO3*, *SLC2A1*, *CACNA1A*, *KCNA1*, *SLC26A4*, *SCN2A*, *RHOBTB2*, *GLDC*, *MECP2*, *PRRT2*, *KCNJ10*, *KCNMA1*, *TBC1D24*, *TMEM151A*, *ATP1A3*, *TMEM151A*, *RHOBTB2*, *ADCY5* and *GNAO1*.

Caffeine was mainly used in patients with a mutation in the ADCY5 gene; not all patients showed remission of symptoms, especially patients with static ADCY5-related cervical dystonia.

All percentages refer to the 97 studies that reported pharmacological treatments. Because multiple therapies were often described within a single publication, the percentages are not mutually exclusive. Non-pharmacological strategies such as trigger avoidance or physical/behavioural therapies were not systematically assessed and are thus excluded from this analysis.

## 4. Discussion

In our systematic review of the literature, it appears that there are 605 paediatric patients described in the literature with paroxysmal dyskinesia. There is a clear prevalence of male patients, a finding that is confirmed in the literature; for example, a recently published study by Huang et colleagues in 2020 [126] confirms the trend of having a clear prevalence in the male sex. Most of the patients described in this systematic review of the literature have paroxysmal kinesigenic dyskinesia. Notably, Kim and colleagues in 2018 [6] describe the highest number of individuals with PKD (paediatric age), with 40 cases. This finding coincides with the literature, according to which [127] PKD is the most common paroxysmal movement disorder. Among the genetic causes that cause PKD, the main one is the *PRRT2* gene, as already described by Chen and colleagues in 2011 [128]. The *PRRT2* gene, in our systematic review, appears to be the gene most involved in the development of paroxysmal dyskinesias that have an underlying genetic cause, contributing to the genesis especially of paroxysmal kinesigenic dyskinesias; we would like to specify that, as for example in the case of the Vaia et al. article from 2023 [26], it is pointed out that episodes of kinesigenic dyskinesia can originate as early as the age of 3 months of life. The most commonly used therapy in paroxysmal dyskinesias is carbamazepine. This finding is confirmed in the literature, where carbamazepine is the most widely used drug especially in paroxysmal dyskinesias kinesigenic from *PRRT2* gene mutation [118,129]. The effectiveness of therapy dramatically affects patients’ quality of life. Although it is not always a data point that is not easily statistically quantifiable, and therefore not integrated into this systematic review, we would therefore like to emphasize that individuals with PKD also present with nonmotor symptoms, which greatly affect quality of life, as well described in this recent article by Asya Ekmen et colleagues in 2023 [130]. Carbamazepine, in general, works well on PKD, although the best results are obtained when the PRRT2 gene is involved. To understand the reasons for this mechanism, we have to remember how sodium blockers act; CBZ/OXC block and reduce neuronal excitability, which underlies neurological manifestations, including PKD [131]. The PRRT2 gene encodes for a protein located at the pre-synaptic level, which interacts with the SNARE complex, regulating neuronal excitability [132].

About the frequency of the types of PD found, after PKD we find PNKDs. The article containing the largest number of PNKDs is that of Masnada et colleagues [110] where 11 individuals with mutation on the *SLC16A2* gene are described who have paroxysmal motor dyskinesias following external stimuli (e.g., a startle) or during a meal. Other genes are implicated in the genesis of PNKDs, including *ADCY5*. One of the genes most implicated in the development of PNKD is the ADCY5 gene. The early diagnosis of paroxysmal dyskinesias related to this gene appears particularly important, as caffeine therapy [133] significantly reduces dyskinetic symptoms (both in cases of PNKD and in the development of other types of paroxysmal dyskinesias). *ADCY5* encodes for a type 5 adenylate cyclase, expressed mainly in the striatum. A2A receptors determine its activation, while D2 dopamine receptors inhibit it. Caffeine has A2A receptors as its main target [134,135]. The cases that are less responsive to therapy among those described in our systematic review involving the ADCY5 gene are, for example, those described by Quazza and colleagues [45], who describe movement disorders associated with the onset of static cervical dystonia; they seem to benefit most from methylphenidate therapy (0.6 mg/kg/day) in one case (PKD, PNKD and nocturnal episodes) and in one case with spontaneous resolution once they reach adulthood (PHD).

Regarding PEDs, of interest is François-Heude et al., 2022 [74] where movement disorders in valine metabolism, caused by *HIBCH* and *ECHS1* deficiencies, are described. However, it is evident from both the literature and our systematic review that one of the genes mainly implicated in PEDs, as well as in other types of PD, is *SLC2A1*. The SLC2A1 gene encodes for GLUT1. The GLUT1 transporter allows glucose to pass through the blood–brain barrier. Its deficiency can therefore cause neurological manifestations such as epilepsy, neurodevelopmental disorders and movement disorders (including PD). The GLUT1 transporter allows the passage of glucose across the blood–brain barrier. Its deficiency can therefore cause neurological manifestations such as epilepsy, neurodevelopmental disorders and movement disorders (including PD) [136]. The ketogenic diet is an excellent solution in patients with this disease, as the brain uses ketones to obtain the energy it needs, instead of glucose, which is prevented from crossing the blood–brain barrier [137]. In the included studies, one of the most suggestive is that of Takahashi and colleagues, from 2020 [34]. Here, patients with the SLC2A1 mutation who have movement disorders and epilepsy are described. Patients on a ketogenic diet showed an improvement in movement disorder and, specifically, PD.

Another gene mentioned in the literature is PNKD. This gene, whose name is evocative for the dyskinesia it refers to, is located on chromosome 9. As described by Harvey and colleagues in 2025 [81], the main triggers in this case are stress and fatigue (but activation by alcohol and coffee is also possible) and transmission is autosomal dominant [138].

PHD deserve a special mention. This group, which constitutes a separate category in paroxysmal dyskinesias, is certainly the rarest and sometimes associated with peculiar genes; the gene most involved in our sample was ADCY5. Law and colleagues [100] describe carbamazepine therapy as effective in 1 patient with PHD; however, the efficacy of therapy appears to be more related to the gene involved in PHD.

Our systematic review has several limitations. In particular, the inclusion criteria for enrolling patients were rather stringent. Some articles of scientific interest may have been excluded; however, we believe this review gives a valid impression and is true to current scientific knowledge regarding paroxysmal dyskinesias in paediatric age.

## 5. Conclusions

The case history of the literature, in the time frame we have taken into consideration, has allowed us to obtain useful information. The first thing to perform is the anamnesis. Here, we will have to pay attention to the movements that are described by the patient and caregivers and any familiarity to movement disorder, and ask the parents if it is possible to make a video of the episodes [139]. Only later, when we have real suspicion of a movement disorder, can we propose genetic analyses. In our sample, as many as 16.5% of patients did not have a definite genetic diagnosis. This could be the starting point for carrying out extended genetic investigations, even beyond the genetic panels for movement disorders, in order to not miss variants that may not have been associated with similar phenotypes up to now.

It is also true, however, as in the case of *PRRT2* paroxysmal kinesigenic dyskinesias, that having positive genetics allows us to go more safely towards a therapy such as carbamazepine or other sodium blockers, to strengthen the clinical suspicion of the referring physician. The same discussion about therapy is valid for other genes, such as *SLC2A1* (ketogenic diet) and *ADCY5* (caffeine). Further genetic and physiopathological studies of paroxysmal dyskinesias will be essential in the future to better understand which therapies can help us interrupt the disabling motor phenomenology and improve the quality of life of these patients.

## Figures and Tables

**Table 1:** Gender distribution of patients with paediatric dyskinesias from studies with available gender data.

	Number	Percentage (%)
Total patients	476	
Male	302	63.4
Female	174	36.6

**Table 2:** Age at onset of paediatric dyskinesias across studies. The table summarizes the descriptive statistics for the reported mean age at onset across studies. Values were standardized to years. Entries reported in days or months were converted accordingly. Studies without usable age data were excluded from the calculation.

**Number of patients with available age data**	601
**Mean (years)**	5.99
**Median (years)**	5.00
**Minimum (years)**	0.027
**Maximum (years)**	17.00
**Standard deviation**	4.77

**Table 3:** Distribution of dyskinesia subtypes among paediatric patients. The table summarizes the total number and percentage of patients diagnosed with different subtypes of paroxysmal dyskinesias across included studies. Subtypes include Paroxysmal Kinesigenic Dyskinesia (PKD), Paroxysmal Non-Kinesigenic Dyskinesia (PNKD), and Paroxysmal Exercise-induced Dyskinesia (PED). All values are based on reported data; missing entries were excluded from percentage calculations.

Type of Dyskinesia	Number of Patients	Percentage (%)
PKD	343	56.8
PNKD	142	23.5
PED	119	19.7

**Table 4:** Genetic and syndromic associations in Paroxysmal Hypnogenic Dyskinesia (PHD).

Gene/Condition	Number of Cases
*ADCY5*	13
*NACC1*	4
*PRRT2*	1
*SLC16A2*	2
*ATP1A3*	1
*DNML1*	1
Brachytelephalangic chondrodysplasia punctata	1

**Table 5:** Frequency of genes associated with paediatric dyskinesias. Percentages refer to patients who have genetically determined paroxysmal dyskinesia. For the “gene” column, if a numeric value preceded a gene (e.g., ‘5 *PRRT2′*), only that number of patients was attributed to the gene. Entries marked as ‘NA’ (not applicable) or “0” were excluded from the genetic analysis. Among the genes causing paroxysmal dyskinesia, the *GCH1* gene is also described, although the number of affected individuals cannot be determined. The raw data and gene count script are available in the Supplementary Material (Supplementary Table S1).

	Count	Percentage (%)
*PRRT2*	231	45.74
*SLC2A1*	72	14.26
*ADCY5*	26	5.15
*ECHS1*	13	2.57
*LMX1B*	13	2.57
*GNAO1*	12	2.38
*SLC16A2*	12	2.38
*RHOBTB2*	11	2.18
*KCNMA1*	10	1.95
*TBC1D24*	9	1.76
*ATP1A3*	8	1.6
*KCNA1*	6	1.19
*TMEM151A*	6	1.17
*CACNA1A*	5	0.98
*HIBCH*	5	0.99
*NACC1*	4	0.79
*PNKD*	4	0.79
*CHRNA4*	3	0.59
*SCN8A*	2	0.39
*DNML1*	2	0.39
*FOXG1*	2	0.39
*KCNJ10*	2	0.39
*SLC26A4*	1	0.20
*SCN2A*	1	0.20
*GLDC*	1	0.20
*FGF14*	1	0.20
*PIGN*	1	0.20
*ANO3*	1	0.20
*PDE2A*	1	0.20
*PDHA1*	1	0.20
*KCNQ2*	1	0.20
*NAA15*	1	0.20
*MECP2*	1	0.20
*NALCN*	1	0.20
*SYNGAP1*	1	0.20
*FARS2*	1	0.20
*NGLY1*	1	0.20

**Table 6:** Chromosomal abnormalities associated with paediatric dyskinesias.

Chromosomal Abnormality	Number of Patients	Percentage (%)
16p11.2 deletion	7	1.39
16p11.2 microduplication syndrome	2	0.39
16p11.2 microdeletion syndrome	1	0.20

## Data Availability

No new data were created or analysed in this study.

## Supplementary Materials

The following supporting information can be downloaded at: https://www.mdpi.com/article/10.3390/jcm14175925/s1, Supplementary Table S1: patients described in this systematic review, with clinical and genetic characteristics. Figure S1: PRISMA 2020 checklist.
